# The Possible Influence of Vitamin D Levels on the Development of Atrial Fibrillation—An Update

**DOI:** 10.3390/nu15122725

**Published:** 2023-06-12

**Authors:** Szymon Graczyk, Arkadiusz Grzeczka, Urszula Pasławska, Pawel Kordowitzki

**Affiliations:** 1 Department of Biological and Veterinary Sciences, Faculty of Basic and Preclinical Sciences, Nicolaus Copernicus University, 87-100 Torun, Poland; graczyk@umk.pl (S.G.);; 2 Department of Biological and Veterinary Sciences, Faculty of Diagnostic and Clinical Sciences, Nicolaus Copernicus University, 87-100 Torun, Poland

**Keywords:** atrial fibrillation, vitamin D deficiency, vitamin D supplementation

## Abstract

Atrial fibrillation (AF) is a severe and most common supraventricular arrhythmia in humans, which, if left untreated or treated ineffectively, can lead to ischemic stroke or heart failure. It has been suggested that serum vitamin D (VitD) deficiency may be one of the critical factors influencing the onset of AF, especially in the period after cardiac surgery, such as coronary artery bypass grafting. Several papers have indicated that VitD supplementation reduces the risk of AF, significantly reducing the proportion of patients between the control and study groups in both the pre- and postoperative periods. Factors that increase the risk of AF from VitD deficiency are also further indicated, and these are age, gender, weight, season or comorbidities. In addition, the cardiodepressive mechanism of VitD is not fully understood; however, it is suggested that it acts through at least two pathways. The first indicates a direct effect of VitD on atrial muscle degradation, while the second is related to the modulation of cardiovascular depression factors. Despite many reports showing correlations between no VitD concentrations on the development of AF, this topic is still widely debated and the results from these papers are still subject to doubt. Therefore, this review aims at describing in detail the problem of correlation between VitD deficiency and the development of AF associated mainly with the postoperative period, i.e., after cardiac surgery, especially pathogenesis, and results of this correlation, taking into account recent studies, limitations and future perspectives. Due to the fact that this is still a topical problem, we believe that the collection of the latest reports and a detailed description of the problem is most appropriate in this case.

## 1. Introduction

Vitamin D (1,25(OH)D) deficiency is seen in various populations around the world regardless of ethnicity [1,2,3,4]. Vitamin D (VitD) status is determined by serum testing and can vary widely depending on age, gender, complexion, season or location. VitD can be ingested in the diet as well as synthesized in a complicated metabolic process involving hydroxylation reactions in two stages. The initiation of VitD synthesis takes place in the skin as a result of UVB radiation. Its precursor is 7-dehydrocholesterol, which is an intermediate of cholesterol synthesis, and its activity depends on the concentration of 7-dehydrocholesterol reductase (DHCR7), so the intensity of preVitD synthesis depends on the concentration of DHCR7 and exposure to sunlight. PreVitD is taken up in the blood by vitamin-D-binding protein (DBP) and transported mostly to the liver. Here, through the action of specific CYP450 hydrolases, such as CYP2D11, CYP2D25 and CYP2R1, another form of VitD, 25-hydroxyvitamin Dt, is obtained through hydroxylation of the carbon ring at position 25 [5]. Of these three hydroxylases, CYP2R1 is the most important, and its activity has also been found in the testes [6]. Subsequently, there is a reuptake, still inactive form of VitD, via DBP transporting it to the kidneys; where, under the action of α-1 hydroxylase, also known as CYP27B1, an actively biological form of VitD (1,25-dihydroxyvitamin Dt) is produced. Activation occurs due to a second hydroxylation at 1 position of the carbon chain [7]. It is well known that the hormonally active form of VitD controls calcium metabolism, but in recent decades, it has been shown to be a very important regulator of other organismal systems. It is difficult to argue with the importance of VitD in the face of numerous papers proving the usefulness of VitD in the treatment of musculoskeletal disorders, as well as its effect on immune function or correlation with overall body condition [8,9,10,11,12]. However, not only the coexistence of musculoskeletal and cardiovascular conditions associated with VitD deficiency is indicated, but also the independent effect of VitD on the cardiovascular system [13]. One of these is atrial fibrillation (AF), which is the most commonly diagnosed arrhythmia nowadays. The worldwide incidence of AF has increased significantly over the past three decades and is now approximately 60 million cases [14]. In addition to the high prevalence of the disease, the costs associated with hospitalizing and treating patients require large amounts of funding. It has been calculated that the resources devoted to AF-related treatment and campaigning range between $6 and $26 billion per year in the US [15]. Despite the financial outlay and treatment strategies developed, this arrhythmia is still associated with a high number of complications related to thromboembolic stroke, progressive congestive heart failure, reduced quality of life or even sudden cardiac death [16]. Its treatment includes rhythm control through the use of antiarrhythmic drugs which prevents its recurrence and also anticoagulant therapy reducing the risk of thromboembolic stroke [17]. Another option is to undergo surgical ablation of the AF initiating center as an alternative to the conventional treatment [18]. Currently, there are two types of AF, the first of which is incidental AF (IAF) that is caused by the chronic effects of profibrogenic factors, while the second arises most often from heart surgery and is referred to as postoperative atrial fibrillation (PoAF).

The purpose of this review will be to identify the relationship and function played by VitD in the pathogenesis of AF, as well as to summarize recent years of research on its supplementation in the context of IAF and PoAF prophylaxis and prevention.

## 2. Pathogenesis of AF and Role of VitD in Its Induction

**Figure 1:**
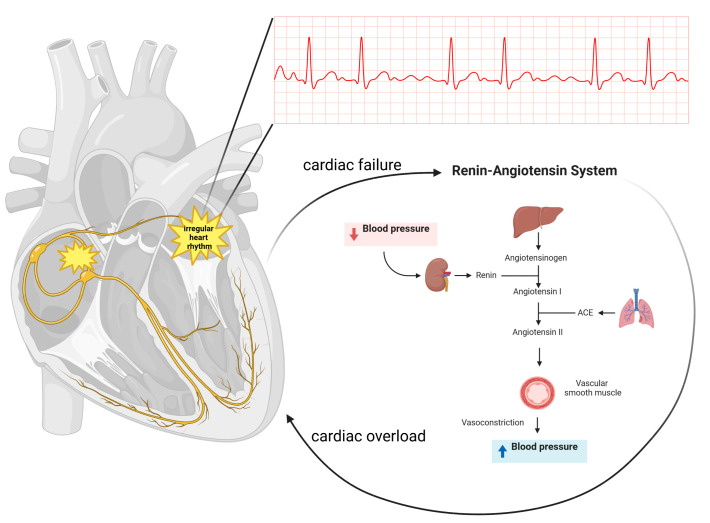
Scheme showing the link between atrial fibrillation and the renin–angiotensin system. The renin–angiotensin–aldosterone system is a widely recognized regulatory axis involved in many processes in the body. It affects, among others, the regulation of blood pressure, and disorders in the system appear to influence the pathogenesis of atrial fibrillation.

The pathogenesis of IAF is not fully understood; however, researchers have identified factors that appear to be critical elements in the development of IAF. Among them are disorders of the renin–angiotensin–aldosterone system (RAAS) (Figure 1), indicators of inflammation and especially C-reactive protein (CPR), fatty acid metabolism and reduced levels of VitD which correlate in some way with the previously mentioned factors [19,20,21,22,23,24,25]. In addition, a decrease in its activity in the blood affects the development of diseases that contribute to IAF. Altogether, it significantly affects the balance of extracellular matrix proteins, namely, collagen of various types which is the structural binder of surrounding tissues. Therefore, the determination of biomarkers of synthesis and degradation, such as carboxy-terminal telopeptide of type I collagen (ICTP) and *N*-terminal propeptide of type III collagen, can indicate atrial structural changes [25,26]. Their labeling in the context of structural changes in the heart is most reasonable due to the fact that type I and type III collagens are most abundant there [27]. However, it should be remembered that the determination of their values in single samples is not fully authoritative because it relates to the degradation and synthesis of collagen in all tissues of the body, so regular determination and development of patterns in a permanent study group allows to determine the actual condition of a particular organ [26]. The exception is when interstitial atrial lesions occur without comorbidities. Moreover, it seems that collagen III biomarkers are much more reliable than collagen I due to their higher increase in plasma [23].

The RAAS is a widely understood regulatory axis involved in many processes in the body. Among other things, it influences the regulation of blood pressure, the body’s water and mineral balance or the secretion of hormones (antidiuretic hormone) [28]. Abnormalities in the functioning of this axis cause serious disorders related to the renal or cardiovascular system [29]. Excessive and prolonged stimulation of the RAAS axis leads to chronic heart failure where angiotensin II plays a key role [30]. It leads to hypertrophy of myocardial cells and hyperproliferation of fibroblasts which result in the accumulation of collagen of various types and the appearance of an interstitial fibrosis [31,32]. In addition, during high RAAS activity, the expression of cardiac receptors for aldosterone is increased which further stimulates angiotensin-converting enzyme in myocardial cells increasing the production of angiotensin II driving the process of structural remodeling of the heart and inducing chronic heart failure [33]. The presence of fibrosis leads to impaired electrical conduction resulting in re-entry loops or focal beats and inducing IAF [34]. VitD is known to be one of the inhibitors of the RAAS axis, so the nuclear VitD receptor (VitDR) present in myocardial cells prevents the above-described changes [35]. Its deficiencies are associated with hypertension, which is dependent on RAAS axis activation [36]. Ozcan et al. 2015 indicated that patients with hypertension with simultaneous VitD deficiency developed IAF [24]. The authors excluded patients with comorbidities, which eliminates the potential role of other diseases in the development of IAF, indicating that the sympathetic nervous system was overstimulated and thus the RAAS axis was hyperactivated. Similar conclusions were reached by Kirchhof et al. (2014), who note the critical role of the RAAS in the development of IAF in hypertensive patients [37]. Contradictory correlations were noted by other authors, suggesting that VitD levels between the control group and the study group with IAF were not significantly different. In addition, they noted reduced angiotensin II levels with significant increases in renin and ICTP. However, it should be noted that the study had a significant number of limitations such as a small group of patients undergoing the study or lack of information on comorbidities or medications taken [25]. Many studies indicate that the use of RAAS axis inhibitors reduces the risk of IAF [31,32,33,34,35]. A study of 47,062 patients indicated that VitD deficiency had no positive correlation with the occurrence of IAF, but reduced levels of VitD significantly reduced the efficacy of RAAS axis inhibitors in the treatment of IAF [38], suggesting that VitD acts as a synergist in inhibiting the RAAS axis, which is consistent with the earlier description of the interaction between VitD and the RAAS axis. 

Inflammatory factors are believed to lead to IAF in various pathological conditions, including through adipose tissue in obese individuals, hypertensive disease, coronary artery disease or autoimmune reactions. In addition, a phenomenon such as “AF begets AF” has been observed, in that proinflammatory factors induce IAF through cardiac remodeling and interstitial fibrosis, and existing IAF further drives the synthesis of proinflammatory cytokines [21]. Structural remodeling occurs mainly through TNF which activates signaling pathways for TGF-B and stimulates myofibroblasts. In addition, inflammation increases the activity of myeloperoxidase II and IX, known as critical extracellular matrix metalloproteinases [39,40,41]. Others are platelet-derived growth factor A (PDGF) and also HSP27 and IL-6 [38,42,43]. To date, little is known about the effects of VitD on inflammatory markers in the context of IAF, but it is known that VitD plays an important role in inhibiting inflammation by acting on most of the inflammatory factors [21,44,45] and also induces the synthesis of anti-inflammatory cytokines [46]. CRP factor is known to be a good and independent indicator of IAF [47]. A correlation has been found between serum VitD levels and the activity of the aforementioned protein in various disease states, including the cardiovascular [48,49,50]. One study found that significantly reduced VitD levels, but still without inflammation, did not induce cardiovascular problems, but reduced VitD levels, along with high CRP levels, increased the likelihood of cardiovascular disease by as much as 2.69 times [50]. Studies using a high intramuscular dose of cholecalciferol VitD in COVID-19 patients indicated a reduced risk of IAF, as an adverse effect to the underlying disease, compared to a group that received a low dose of oral VitD in the form of alfacalcidol [51]. Although there is much evidence that VitD interacts closely with inflammatory factors, research is still needed to determine their role in the occurrence and, more importantly, in predicting IAF.

Obesity, as well as IAF, is a highly topical and growing problem. Figures provided by the WHO for 2022 indicate that the number of obese people has exceeded more than 1 billion people worldwide. Unfortunately, this condition brings with it a number of unpleasant consequences, including an increasing trend in the incidence of various diseases which seems most alarming [52]. Among them, cardiovascular diseases such as heart failure, IAF, hypertension and coronary heart disease account for a large percentage [53]. The development of IAF on a fatty background is related to the deposition of nonesterified fatty acids (NEFAs) in epicardial adipose tissue (EAT) leading, as in previous cases, to structural remodeling of the heart manifested by enlargement and morphological changes of the left atrium [54]. The remodeling includes NEFA-induced myocardial fibrosis. In addition, the already growing EAT activates immune responses, mainly through T cells [55] and produces fibrotic factors, such as reactive oxygen species, proinflammatory cytokines, metalloproteinases and TGF-beta1 [56,57,58]. Moreover, it has been proven that EAT has a paracrine effect on the myocardium through the synthesis of adipofibronectin-activin A, which has a fibrogenic effect on the atrial wall [59], thus, together with the above factors, disrupting electrical conduction increasing the likelihood of IAF. To prevent excessive fat accumulation in tissues, fatty-acid-binding proteins (FABPs) exist in the body in different isoforms. In cardiovascular disease, adipose-FABP (A-FABP) and heart-FABP (H-FABP) are the most important, and they counteract EAT deposition by transporting fat molecules to intracellular sites [57]. H-FABPs, found in large amounts intracellularly, bind NEFAs from the surface of cardiomyocytes and transport them into the cell to then be involved in the citric acid cycle in the mitochondria [60]. Increasing damage and changes in the myocardium cause H-FABP to be released into the blood, thereby reducing the ability of cardiac muscle cells to bind NEFA and use it as an energy source [61,62]. Therefore, it is believed that plasma H-FABP levels may serve as a potential marker in predicting cardiovascular diseases, including IAF [61]; however, Odeh et al. indicate that each of the FABP isoforms may play a role in the pathogenesis of IAF and serve as a potential diagnostic biomarker. Although a study describing the direct effects and correlations between VitD and anthropometric indices in obese individuals has yet to emerge, the data available in the literature point to its potential role in the induction of IAF in this weight group. First, there are papers summarizing several recent years of research indicating reduced VitD levels in obese individuals, thus predisposing them to a number of diseases [63,64,65]. Second, it was indicated that obesity and waist circumference were significantly associated with a higher incidence of IAF [66], and each 1 k/m^2^ increase in BMI increases the likelihood of IAF by 4.7% [67]. The third is that factors secreted and regulated by adipose tissue affect the molecular mechanisms that induce atrial remodeling [68,69]. Finally, the fourth, overweight is associated with activation of the RAAS and a systemic response in the form of hypertensive states, as well as local responses related to the profibrotic effects of angiotensin II and aldosterone, as seen in the myocardium [70,71]. Although no data have yet been published on the effect of VitD on IAF induction in individuals, there is plenty of evidence that it may be one of the more important regulators of this pathomechanism; hence, it is important to focus future research in this direction.

There are several hypotheses and evidence for the pathomechanism of PoAF, but the exact cause has not yet been defined. It is believed that it is not a single mechanism but a series of processes collectively triggering PoAF. The condition itself carries serious consequences in the form of stroke, thromboembolic diseases or sudden cardiac death [72]. Moreover, it affects between 15 and 60% of the population after cardiac surgery generating increased medical costs and hospital stay [73,74]. Induction of PoAF is associated with a short postoperative period, reaching up to 6 days [75], with the most common cases of PoAF reported between 24 and 72 h [76], which is also consistent with peak levels of leukocytes or inflammatory markers [77]. Considering the pathophysiology of IAF from VitD deficiency, it appears that POAF induction has a very similar basis. The majority of patients showed increased activity of critical proinflammatory factors, i.e., IL-1, IL-6, TNF-alpha and CRP [78], but it is worth mentioning that the activity of these cytokines was not elevated in all PoAF cases [77,79]. In addition to generalized, subclinical inflammation, pericardial fluid effusion occurs due to cardiac surgery, contributing to local inflammation affecting cardiac tissue. Moreover, despite the availability of the best surgical technologies, tissue irritation in the case of CABG or ablation of ectopic foci, as well as other cardiac procedures, results in local inflammation, remodeling the microenvironment of the atrial muscle, which consequently induces cardiomyocyte apoptosis and electrical conduction disturbances [72,74]. VitD is one of the regulators against developing inflammation; hence, it is believed that individuals undergoing cardiac surgery with concomitant VitD deficiency are more likely to develop PoAF, and VitD supplementation may prevent PoAF in most patients [80,81,82]. It has also been assumed that activation of the sympathetic nervous system also contributes to PoAF by altering atrial refractoriness and also promoting ectopic activity through noradrenergic stimulation [83]. It appears that VitD deficiency may also play a role in this case. Although there have been no studies to date determining the effect of VitD on PoAF when activating the sympathetic nervous system, a study on a group of young, healthy individuals confirmed that VitD supplementation in young people with deficiency modulates the sympathetic nervous system, which may prevent the onset of heart disease in the future [84]. However, further studies are required to determine this effect on the group entering cardiac surgery. Finally, another pathway for the induction of PoAF from non-rebound VitD may be the activation of the RAAS axis, which is also linked to sympathetic nervous system stimulation. In addition, the use of aldosterone as a biomarker for the prevention of PoAF may serve as a good prognostic indicator for those burdened with this condition [85,86]. As previously mentioned, VitD is one of the inhibitors of this axis, and its deficiencies may be associated with excessive atrial fibrosis and the formation of ectopic foci and re-entry loops. It has been documented that VitD supplementation inhibits the development of PoAF in some patients with excessive RAAS activation [87,88]. PoAF on the background of VitD deficiency is multifactorial, and there are many indications that it acts through IAF mechanisms, but it seems that the critical point is the level of VitD at the time of entering cardiac surgery, since such procedures induce high stress and mobilize the body to counteract human-induced changes. This mobilization is greater if lower the level of VitD recorded before the procedure [89,90], where its inhibiting effects on the RAAS and inflammation are significantly regressed. For this reason, determining VitD levels before cardiac surgery and possible supplementation is quite important.

## 3. Relationship of VitD Deficiency and Supplementation to the Risk of PoAF and IAF

The IAF and PoAF that we are discussing, which are caused by other processes described above, differ both in terms of frequency, recorded VitD levels and the impact of supplementation. In this section, we intend to discuss, separately for IAF and PoAF, based on the available papers, the probability of AF occurrence in VitD deficiency, the VitD concentration that was most often associated with AF, the most effective supplementation dose and timing and the position of meta-analyses that have been conducted over the years to evaluate the effect of VitD on AF occurrence.

### 3.1. PoAF

The vast majority of the papers reviewed below addressed the effect of VitD deficiency and/or supplementation on the occurrence of PoAF in the context of coronary artery bypass grafting (CABG) [91,92,93,94]. During the hospitalization period following this procedure, patients often develop AF with varying frequency. Most commonly, a range of 20–30% of patients is indicated, but much lower frequencies in the 12–16% range have also been reported [20]. This is directly related to the level of VitD deficiency. As researchers point out, as a result of the preoperative stress the patient is experiencing, there can be a sudden drop in plasma VitD levels [80,95]. VitD deficiency, which is one of the elements of the pathomechanism of PoAF, can be profound or moderate. Therefore, studies most often use classifications of VitD levels: deficiency to [25(OH)D2] < 20 ng/mL; insufficiency to 20 ng/mL < [25(OH)D2] <30 ng/mL; normal to [25(OH)D2] > 30 ng/mL. AF has been reported in both deficient, insufficiency and normal states, but most often the average VitD level at which AF was reported was below 20 ng/mL [1,3,9,10]. Gode et al., whose PoAF occurred with a VitD level of 9.0 ± 5.0 ng/mL, detailed that 66.6% with PoAF had levels below 10 ng/mL, and 33.4% had levels of 10–30 ng/m [82]. Moreover, with similar data (PoAF occurring at a VitD concentration of 7.49 ± 3.81), a cutoff point of 7.65 ng/mL was set, which had a 60% sensitivity and 64% specificity [11]. Therefore, also the treatment of the “deficiency” group is more effective than the treatment of the “insufficiency” group [96]. In the study, a difference of 11% PoAF was obtained between the “deficiency” treatment group (18%) and the “insufficiency” control group (29%) using 50,000 IU 48 h before surgery. Equally satisfactory results were obtained by supplementing with 600,000 IU 5 days before surgery [97]. However, the best results were obtained using doses adjusted to the level of VitD requirement 48 h before surgery [93]. Patients with “deficiency” received 300,000 IU and “insufficiency” received 150,000 UI, resulting in a 15.52% difference between the study and control groups [93]. Perhaps, this treatment reduced the number of patients who were exposed to an excessively high-toxic dose of VitD.

The overwhelming number of studies point to an association between VitD and PoAF. However, without overlooking studies that prove the opposite, there is evidence of an inverse correlation between VitD and PoAF [98,99]. According to some, higher levels of VitD should increase the likelihood of PoAF [94,100]. One should also consider whether VitD is an independent predictor of PoAF. However, it should be noted that in a study that debunks this value of VitD concentration, the PoAF+ group nevertheless has a high level of VitD (19.5 ng/mL) [91]. 

Using the conclusions of meta-analyses devoted to this topic, the above uncertainties can be partially answered. Of the seven meta-analyses that focused on the level of VitD and its relationship to PoAF frequency, six indicated a significant effect of the VitD concentration [43,101,102,103,104,105,106]. One contrary paper also clustered papers on IAF and did not distinguish between them, so we decided to treat the aforementioned four as prognostic [43].

### 3.2. IAF

Available attempts to determine the frequency of IAF involve different study groups, frequently over many years, often grouping patients with comorbidities [24,107,108,109,110]. Therefore, the frequency of IAF more than once ranges in probability from 1.4% to 27.59% [111,112,113]. The increased risk of AF consists, for example, of cardiovascular diseases such as hypertension or valvular disease [19,24,107]. Moreover, in at-risk groups, lower VitD values contributed to an increased incidence of IAF [107]. In addition, as VitD deficiency worsened, which is also associated with elevated PTH levels, the incidence of AF also increased [114]. To determine the level of deficiency, observational studies use a similar classification to PoAF. Again, [25(OH)D2] values <20 ng/mL are predictive of AF incidence [19,115,116]. Deficient values in one study were associated with 17.2% of AF in the group, while normal values were 10.9% [117]. In a study group from China, [25(OH)D2] values <20 ng/mL led to a twofold higher risk of AF [115]. The values themselves vary widely and are likely influenced by comorbidities. For example, the values that occur in cardiovascular diseases (valvular disease, heart failure and hypertension) and are associated with AF are respectively 9.24 + 7.39 ng/mL; 11.05 ng/mL; 16.8 + −6.5 [19,24,111]. However, cutoff values for new-onset AF have been found to be much higher, at 16.50 ng/mL or 22.5 ng/mL, according to some authors [24,111]. In healthy populations, on the other hand, VitD values associated with AF are, for example, 19.6 ± 7.4 ng/mL and −18.5 ± 10.3 ng/mL [115,116]. In the case of VitD supplementation, very long periods (more than 5 years) did not result in a reduction in AF risk [108]. Another study, on the other hand, showed the effectiveness of VitD supplementation over a period of 6 months [107].

Although most studies devoted to observing IAF do not show a correlation between VitD concentration and AF frequency, three out of four meta-analyses devoted to this topic show a significant correlation [43,103,118,119].

## 4. Why Is It So Difficult to Determine the Effect of VitD on AF Incidence?—Limitations

Despite high hopes for the potential properties of VitD in reducing the likelihood of AF episodes, there is still no clear answer to the question of the VitD utility in this regard [101,103,105,120,121,122]. This has to do with the limitations posed to researchers by the substance itself, whose metabolism is extensive and affects numerous tissues through interactions with nuclear and membrane cell receptors [123,124,125,126]. In addition, VitD interacts with hormones that globally affect the entire body [127,128]. There are also technical and presumptive limitations related to the study group, the study methodology, the detection of AF and the determination of its nature, as well as how VitD is supplemented.

VitD is a substance that is subject to seasonal variations [96,129]. Therefore, depending on the season, we can expect different levels of VitD, and thus, there may be difficulties in adjusting the appropriate dose of supplementation [93,103,108,130]. In some cases, additional supplementation that was not included in the medical records could not be ruled out [107,131]. Depending on the biosynthesis and administration in food or supplements, VitD affects the degree of calcium resorption from the diet and also stimulates the release of calcium and phosphate from bone, so it is a very important element in maintaining calcium–phosphate homeostasis and musculoskeletal health [124]. Although researchers are not sure which form, VitD2 or VitD3, supplemented most strongly influences the elevation of serum VitD concentrations, they collectively assert that many factors model the absorption of VitD from the gastrointestinal tract [132,133]. For VitD contained in food to be utilized, it must be released from the food. First and foremost, the presence, amount and type of fats are important for VitD, due to its hydrophobic properties [134]. The influence of fiber, age, degree of obesity and vitamin status is also indicated [135,136]. The degree of hydration of vitamin D used for supplementation is also important. It has been indicated that hydroxylated vitamin D3, i.e., (25(OH)D3), has a higher potential to raise plasma VitD concentrations than nonhydrated forms [132]. Thus, it is important to be mindful of the actual amount of VitD intake, especially since there are reports of cardiotoxic and proarrhythmic effects of too high VitD concentrations [113]. In turn, calcium deficiency and VitD are known to cause secondary hyperparathyroidism, which results in loss of bone mass and, most importantly, is associated with a higher frequency of AF [129]. Thus, the measurement of PTH levels is not only one of the parameters that clarifies the knowledge of VitD status but also the risk of AF [111]. One study indicated that in the PTH-deficient group, the frequency of AF (16.4 per 1000 person-years) was lower than in the group with PTH deficiency and VitD (20.3 per 1000 person-years) [114]. Therefore, we believe that papers on VitD that do not take PTH levels into account are less reliable [96,110,114,137]. Especially since most often VitD serum levels are measured at a single time point, making it difficult to determine the duration of VitD deficiency [111]. Age and gender should also be taken into account, as these two characteristics determine different values of VitD and PTH in the body [138]. This is well demonstrated in each age group and results in VitD levels being much higher in men, while PTH hormone levels are much higher in women [138]. It is also worth noting that calcium levels, according to studies, are significantly higher in men, which is important because of the importance of this micronutrient in achieving excitability potential, by cardiac muscle cells. Therefore, some studies in which calcium was administered in addition to VitD cannot be expected to show the real effect of VitD, especially since the interaction between the two—VitD and calcium—is still not fully understood [112,139].

It is also necessary to discuss the limitations associated with the study group. There are papers that appear in which the limitation is still the small size of the study group as well as the very large size of the study group which is difficult to systematize [96,113,140]. Therefore, it is currently more difficult and important to complete a representative group that will apply to a random patient [25,107,110]. The difference in skin pigmentation is significant enough to influence the results of the study, thus obscuring important conclusions [111,141]. Therefore, studies on Caucasian populations, a complexion-diverse study group from Turkey, or results obtained from measurements among veterans are difficult to transfer to a random patient [25,107,110,111]. Finally, analyzing patients with comorbidities, such as hypertension, diabetes mellitus and obesity, who in addition use medications, is an important factor limiting the usefulness of the results, since these entities are factors that increase the occurrence of AF regardless of the level of VitD [25,97,113,142]. Randomized, double-blinded clinical trials have advantages over other studies that present results from selected patients because they are subject to greater control than retrospective studies and are closer to the randomized patient [97]. On the other hand, when analyzing data over several years that may be randomized, the questionnaires and medical records used may be incomplete or unstructured due to changing standards over time [119]. In addition, the risk of bias, unexpected confounders or overly cursory recording of events relevant to the study cannot be excluded.

The onset of AF is associated with nonspecific symptoms, such as shortness of breath, sweats, fainting, dizziness and rapid fatigue [143]. AF can also occur incidentally or be provoked by a cardiac procedure performed [94,142]. This causes many moments of AF to go unnoticed and often leaves no serious consequences, but undiagnosed AF is an important limitation in studying the impact of VitD deficiency. Therefore, ECG is used to detect AF [143]. However, some authors have focused only on the period of hospitalization or used medical records, which are not always perfect [87,110]. In particular, those AFs that are paroxysmal and asymptomatic pose difficulties; however, they are those that make up a significant portion of the total, so knowing their frequency would be very valuable [94,108,144]. One solution is to use Holter monitoring during hospitalization and follow-up examinations after leaving the hospital, which would allow continuous observation of the heart rhythm [91,93]. This would be particularly appropriate in studies focusing on the postoperative AF [82].

## 5. Future Perspectives and Conclusions

Assuming a positive effect of higher concentrations of VitD, future research should focus on determining an appropriate dose that takes into account the needs of the individual patient. According to the study, the timing of perioperative administration of the vitamin is also important. In turn, another part of the study should clarify how long persistent VitD deficiency or insufficiency is a threshold for promoting IAF and PoAF. In addition, in order to assess the exact impact and correlations between VitD deficiency and the induction of IAF and PoAF, it will be important to compare it with factors involved in the pathomechanism of these two conditions such as IL-1, IL-6, TNF-alpha, metalloproteinases and their inhibitors, biomarkers of collagen I and III synthesis and degradation, hs-CRP, NEFA, FABP isoforms as well as angiotensin II and aldosterone. Prospective, randomized, double-blinded, large clinical trials with a high degree of control over the study group and VitD level are needed to complete the knowledge on this topic. 

In the current review, we identified potential mechanisms during which VitD deficiency enhances the induction of IAF as well as PoAF. Both types of arrhythmias are multifactorial in nature, certainly not yet fully described. Before the initiation of IAF in the heart, there are gradual changes indicative of interstitial fibrin deposition, hypertrophy and partial necrosis of cardiomyocytes resulting in disruption of cardiac electrical conduction and isolation of ectopic foci and formation of re-entry loops. Such induced remodeling of cardiac tissue will lead to the occurrence of IAF within a certain time. Profibrotic effects are based on metabolites of RAAS (angiotensin II, aldosterone), fatty compounds and their binding proteins (NEFA, FABP) and also proinflammatory factors (IL-1, IL6 and TNF-alpha). Although AF is divided into incidental and postoperative, the latter is triggered by similar pathways, but the role of VitD and its level in the blood seems to be of greatest importance shortly before cardiac surgery, due to the fact that the lower its activity, the factors described above act even more strongly, leading to the induction of PoAF in a short period of time. In addition, the stress of the procedure activates the sympathetic nervous system which only intensifies the whole pathomechanism of AF.

According to clinical and observational studies, VitD levels can affect the incidence of AF. Moreover, the risk of AF has been shown to increase depending on the degree of deficiency. Although not all studies have shown that VitD is an independent factor, the amount of evidence supports correlations between VitD and AF (Table 1). Therefore, the dose and timing of VitD supplementation should be carefully determined for the patient, as this can significantly reduce the number of patients who develop PoAF with IAF.

## Figures and Tables

**Table 1:** Table showing a summary of studies regarding vitamin D supplementation.

Author [Ref.]	Country	Study Design	Study Population	Patients (n)	Age (yrs)	Gender (Males)	State of VitD	Dose of VitD	Endpoint Considered	Major Findings
[108]	USA	Randomized Double-blind Placebo-controlled	Randomized	25,119	66.7	49.2%	n/d	2000 IU	Incidents of AF	VitD supplementation had no significant effect.
[107]	USA	Case-controlled	Veterans	39,845	57.5	63.1%	Deficiency (25-OH) D <20 ng/mL / Insufficiency 20 <(25-OH) D <30 ng/mL	n/d	Incidents of AF	(25-OH) D >20 ng/mL with VitD for ≥6 months associated with lower AF risk.
[114]	-	Prospective study	Older adults	2481	76.0	-	Deficiency (25-OH) D <20 ng/mL / 20 < Insufficiency <30 ng/mL	n/d	Incidents of AF	Treatment of VitD deficiency may contribute to lower the risk of AF.
[91]	Cyprus	Retrospective study	CABG surgery	128	67.6	85.4%	Present POAF–19.9 ± 6.1 (19.5) ng/mL Absent PAOF-26 ± 8.2 (26.4) ng/mL	n/d	New-onset postop AF	VitD level was not an independent predictor for POAF.
[97]	-	Randomized Prospective study	CABG surgery	196	59.29	70.0%	14.43 ng/mL	600 000 IU 5 days before surgery	POAF during the first 5 days after CABG surgery	VitD supplementation reduces the incidence of POAF.
[94]	Germany	Prospective cohort study	CABG surgery	201	66.6	84.6%	Deficiency 17.7 ng/mL	-	New-onset postop AF	Elevated 25(OH)D concentration and VitD supplementation rather revealed an increased OR for POAF.
[93]	-	Randomized Double-blind	CABG surgery	116	65.05	25%	Deficiency 10.77 ± 3.21 ng/mL / Insufficiency 25.13 ± 3.45 ng/mL	150 000 IU / 300 000 IU 48 h before surgery	New-onset postop AF	Preoperative short-term high-dose VitD supplementation significantly preventive of POAF.
[87]	Cyprus	Case-controlled	CAGB surgery	328	63.8	43.7%	Deficiency 11.4 ± 4.9 ng/mL / Insufficiency 24.6 ± 3.7 ng/mL	50 000 IU 48 h before surgery	New-onset postop AF	VitD administered 48 h before the operation reduces the risk of PoAF in the deficiency group but not in the insufficiency group

## Data Availability

Not applicable.

## References

[1] Duarte C., Carvalheiro H., Rodrigues A.M., Dias S.S., Marques A., Santiago T., Canhão H., Branco J.C., da Silva J.A.P. (2020). Prevalence of vitamin D deficiency and its predictors in the Portuguese population: A nationwide population-based study. Arch. Osteoporos..

[2] Mogire R.M., Mutua A., Kimita W., Kamau A., Bejon P., Pettifor J.M., Adeyemo A., Williams T.N., Atkinson S.H. (2020). Prevalence of Vitamin D Deficiency in Africa: A Systematic Review and Meta-Analysis. Lancet Glob. Health.

[3] Siddiqee M.H., Bhattacharjee B., Siddiqi U.R., MeshbahurRahman M. (2021). High prevalence of vitamin D deficiency among the South Asian adults: A systematic review and meta-analysis. BMC Public Health.

[4] Cashman K.D. (2020). Vitamin D Deficiency: Defining, Prevalence, Causes, and Strategies of Addressing. Calcif. Tissue Int..

[5] Saponaro F., Saba A., Zucchi R. (2020). An Update on Vitamin D Metabolism. Int. J. Mol. Sci..

[6] Cheng J.B., Motola D.L., Mangelsdorf D., Russell D. (2003). De-Orphanization of Cytochrome P450 2R1. J. Biol. Chem..

[7] Christakos S., Ajibade D.V., Dhawan P., Fechner A.J., Mady L.J. (2010). Vitamin D: Metabolism. Endocrinol. Metab. Clin. N. Am..

[8] Cantorna M.T., Zhu Y., Froicu M., Wittke A. (2004). Vitamin D Status, 1, 25-Dihydroxyvitamin D3, and the Immune System. Am. J. Clin. Nutr..

[9] Aranow C. (2011). Vitamin D and the Immune System. J. Investig. Med..

[10] Guillot X., Semerano L., Saidenberg-Kermanac’h N., Falgarone G., Boissier M.-C. (2010). Vitamin D and Inflammation. Jt. Bone Spine.

[11] Prietl B., Treiber G., Pieber T.R., Amrein K. (2013). Vitamin D and Immune Function. Nutrients.

[12] Mathieu C., Gysemans C., Giulietti A., Bouillon R. (2005). Vitamin D and Diabetes. Diabetologia.

[13] Yang S., Zhi H., Sun Y., Wang L. (2022). Circulating Vitamin D Levels and the Risk of Atrial Fibrillation: A Two-Sample Mendelian Randomization Study. Front. Nutr..

[14] Elliott A.D., Middeldorp M.E., Van Gelder I.C., Albert C.M., Sanders P. (2023). Epidemiology and modifiable risk factors for atrial fibrillation. Nat. Rev. Cardiol..

[15] Mh K., Ss J., Bc C., Mr D., Kl S. (2011). Estimation of Total Incremental Health Care Costs in Patients With Atrial Fibrillation in the United States. Circ. Cardiovasc. Qual. Outcomes.

[16] Pastori D., Menichelli D., Violi F., Pignatelli P., Lip G.Y.H. (2020). The Atrial fibrillation Better Care (ABC) pathway and cardiac complications in atrial fibrillation: A potential sex-based difference. The ATHERO-AF study. Eur. J. Intern. Med..

[17] January C.T., Wann L.S., Alpert J.S., Calkins H., Cigarroa J.E., Cleveland J.C., Conti J.B., Ellinor P.T., Ezekowitz M.D., Field M.E. (2014). 2014 AHA/ACC/HRS Guideline for the Management of Patients with Atrial Fibrillation: Executive Summary: A Report of the American College of Cardiology/American Heart Association Task Force on Practice Guidelines and the Heart Rhythm Society. Circulation.

[18] Calkins H., Brugada J., Packer D.L., Cappato R., Chen S.-A., Crijns H.J., Damiano R.J., Davies D.W., Haines D.E., Haissaguerre M. (2007). HRS/EHRA/ECAS Expert Consensus Statement on Catheter and Surgical Ablation of Atrial Fibrillation: Recommendations for Personnel, Policy, Procedures and Follow-Up. A report of the Heart Rhythm Society (HRS) Task Force on Catheter and Surgical Ablation of Atrial Fibrillation Developed in partnership with the European Heart Rhythm Association (EHRA) and the European Cardiac Arrhythmia Society (ECAS); in collaboration with the American College of Cardiology (ACC), American Heart Association (AHA), and the Society of Thoracic Surgeons (STS). Endorsed and Approved by the governing bodies of the American College of Cardiology, the American Heart Association, the European Cardiac Arrhythmia Society, the European Heart Rhythm Association, the Society of Thoracic Surgeons, and the Heart Rhythm Society. Europace.

[19] Demir M., Uyan U., Melek M. (2014). The Effects of Vitamin D Deficiency on Atrial Fibrillation. Clin. Appl. Thromb..

[20] Galea R., Cardillo M.T., Caroli A., Marini M.G., Sonnino C., Narducci M.L., Biasucci L.M. (2014). Inflammation and C-Reactive Protein in Atrial Fibrillation: Cause or Effect?. Tex. Heart Inst. J..

[21] Hu Y.-F., Chen Y.-J., Lin Y.-J., Chen S.-A. (2015). Inflammation and the pathogenesis of atrial fibrillation. Nat. Rev. Cardiol..

[22] Jabati S., Fareed J., Liles J., Otto A., Hoppensteadt D., Bontekoe J., Phan T., Walborn A., Syed M. (2018). Biomarkers of Inflammation, Thrombogenesis, and Collagen Turnover in Patients with Atrial Fibrillation. Clin. Appl. Thromb..

[23] Odeh A., Dungan G.D., Darki A., Hoppensteadt D., Siddiqui F., Kantarcioglu B., Fareed J., Syed M.A. (2022). Collagen Remodeling and Fatty Acid Regulation Biomarkers in Understanding the Molecular Pathogenesis of Atrial Fibrillation. Clin. Appl. Thromb. Hemost.

[24] Ozcan O.U., Gurlek A., Gursoy E., Gerede D.M., Erol C. (2015). Relation of vitamin D deficiency and new-onset atrial fibrillation among hypertensive patients. J. Am. Soc. Hypertens..

[25] Patel D., Druck A., Hoppensteadt D., Bansal V., Brailovsky Y., Syed M., Fareed J. (2020). Relationship between 25-Hydroxyvitamin D, Renin, and Collagen Remodeling Biomarkers in Atrial Fibrillation. Clin. Appl. Thromb. Hemost..

[26] Duprez D.A., Heckbert S.R., Alonso A., Gross M.D., Ix J.H., Kizer J.R., Tracy R.P., Kronmal R., Jacobs D.R. (2018). Collagen Biomarkers and Incidence of New Onset of Atrial Fibrillation in Subjects with No Overt Cardiovascular Disease at Baseline: The Multi-Ethnic Study of Atherosclerosis. Circ. Arrhythmia Electrophysiol..

[27] Mays P.K., Bishop J.E., Laurent G.J. (1988). Age-related changes in the proportion of types I and III collagen. Mech. Ageing Dev..

[28] Fournier D., Luft F.C., Bader M., Ganten D., Andrade-Navarro M.A. (2012). Emergence and evolution of the renin-angiotensin-aldosterone system. J. Mol. Med..

[29] Atlas S.A. (2007). The Renin-Angiotensin Aldosterone System: Pathophysiological Role and Pharmacologic Inhibition. J. Manag. Care Pharm..

[30] Sayer G., Bhat G. (2014). The Renin-Angiotensin-Aldosterone System and Heart Failure. Cardiol. Clin..

[31] Weber K.T. (1997). Extracellular Matrix Remodeling in Heart Failure. Circulation.

[32] Baker K.M., Aceto J.F. (1990). Angiotensin II stimulation of protein synthesis and cell growth in chick heart cells. Am. J. Physiol. Circ. Physiol..

[33] Harada E., Yoshimura M., Yasue H., Nakagawa O., Nakagawa M., Harada M., Mizuno Y., Nakayama M., Shimasaki Y., Ito T. (2001). Aldosterone Induces Angiotensin-Converting-Enzyme Gene Expression in Cultured Neonatal Rat Cardiocytes. Circulation.

[34] Everett T.H., Olgin J.E. (2007). Atrial fibrosis and the mechanisms of atrial fibrillation. Heart Rhythm..

[35] Al Mheid I., Patel R.S., Tangpricha V., Quyyumi A.A. (2013). Vitamin D and cardiovascular disease: Is the evidence solid?. Eur. Heart J..

[36] Vimaleswaran K.S., Berry D.J., Lu C., Tikkanen E., Pilz S., Hiraki L.T., Cooper J.D., Dastani Z., Li R., Houston D.K. (2013). Causal Relationship between Obesity and Vitamin D Status: Bi-Directional Mendelian Randomization Analysis of Multiple Cohorts. PLoS Med..

[37] Kirchhof P., Fabritz L. (2014). Of hammers and screws: Renin-angiotensin-aldosterone system inhibition to prevent atrial fibrillation in patients with hypertension. Eur. Heart J..

[38] Turin A., Bax J.J., Doukas D., Joyce C., Lopez J.J., Mathew V., Pontone G., Shah F., Singh S., Wilber D.J. (2018). Interactions Among Vitamin D, Atrial Fibrillation, and the Renin-Angiotensin-Aldosterone System. Am. J. Cardiol..

[39] Khatib R., Joseph P., Briel M., Yusuf S., Healey J. (2013). Blockade of the renin–angiotensin–aldosterone system (RAAS) for primary prevention of non-valvular atrial fibrillation: A systematic review and meta analysis of randomized controlled trials. Int. J. Cardiol..

[40] Chaugai S., Meng W.Y., Sepehry A. (2016). Effects of RAAS Blockers on Atrial Fibrillation Prophylaxis: An Updated Systematic Review and Meta-Analysis of Randomized Controlled Trials. J. Cardiovasc. Pharmacol. Ther..

[41] Healey J.S., Baranchuk A., Crystal E., Morillo C.A., Garfinkle M., Yusuf S., Connolly S.J. (2005). Prevention of Atrial Fibrillation with Angiotensin-Converting Enzyme Inhibitors and Angiotensin Receptor Blockers. J. Am. Coll. Cardiol..

[42] Schneider M.P., Hua T.A., Böhm M., Wachtell K., Kjeldsen S.E., Schmieder R.E. (2010). Prevention of Atrial Fibrillation by Renin-Angiotensin System Inhibition: A Meta-Analysis. J. Am. Coll. Cardiol..

[43] Huang W.-L., Yang J., Yang J., Wang H.-B., Yang C.-J., Yang Y. (2018). Vitamin D and new-onset atrial fibrillation: A meta-analysis of randomized controlled trials. Hell. J. Cardiol..

[44] Liew R., Khairunnisa K., Gu Y., Tee N., Yin N.O., Naylynn T.M., Moe K.T. (2013). Role of Tumor Necrosis Factor-α in the Pathogenesis of Atrial Fibrosis and Development of an Arrhythmogenic Substrate. Circ. J..

[45] Rudolph V., Andrié R.P., Rudolph T.K., Friedrichs K., Klinke A., Hirsch-Hoffmann B., Schwoerer A.P., Lau D., Fu X., Klingel K. (2010). Myeloperoxidase acts as a profibrotic mediator of atrial fibrillation. Nat. Med..

[46] Canning M.O., Grotenhuis K., De Wit H., Ruwhof C., Drexhage H.A. (2001). 1-alpha,25-Dihydroxyvitamin D3 (1,25(OH)(2)D(3)) hampers the maturation of fully active immature dendritic cells from monocytes. Eur. J. Endocrinol..

[47] Hatzinikolaou-Kotsakou E., Tziakas D., Hotidis A., Stakos D., Floros D., Papanas N., Chalikias G., Maltezos E., Hatseras D.I. (2006). Relation of C-Reactive Protein to the First Onset and the Recurrence Rate in Lone Atrial Fibrillation. Am. J. Cardiol..

[48] Shea M.K., Booth S.L., Massaro J.M., Jacques P.F., D’Agostino R.B., Dawson-Hughes B., Ordovas J.M., O’Donnell C.J., Kathiresan S., Keaney J.F. (2007). Vitamin K and Vitamin D Status: Associations with Inflammatory Markers in the Framingham Offspring Study. Am. J. Epidemiol..

[49] Eleftheriadis T., Antoniadi G., Liakopoulos V., Stefanidis I., Galaktidou G. (2012). Inverse association of serum 25-hydroxyvitamin D with markers of inflammation and suppression of osteoclastic activity in hemodialysis patients. Iran. J. Kidney Dis..

[50] Li Q., Dai Z., Cao Y., Wang L. (2019). Association of C-reactive protein and vitamin D deficiency with cardiovascular disease: A nationwide cross-sectional study from National Health and Nutrition Examination Survey 2007 to 2008. Clin. Cardiol..

[51] Sarhan N., Warda A.E.A., Sarhan R.M., Boshra M.S., Mostafa-Hedeab G., Alruwaili B.F., Ibrahim H.S.G., Schaalan M.F., Fathy S. (2022). Evidence for the Efficacy of a High Dose of Vitamin D on the Hyperinflammation State in Moderate-to-Severe COVID-19 Patients: A Randomized Clinical Trial. Medicina.

[52] Włodarczyk M., Nowicka G. (2019). Obesity, DNA Damage, and Development of Obesity-Related Diseases. Int. J. Mol. Sci..

[53] Ortega F.B., Lavie C.J., Blair S.N. (2016). Obesity and Cardiovascular Disease. Circ. Res..

[54] Alpert M.A., Omran J., Bostick B.P. (2016). Effects of Obesity on Cardiovascular Hemodynamics, Cardiac Morphology, and Ventricular Function. Curr. Obes. Rep..

[55] Haemers P., Hamdi H., Guedj K., Suffee N., Farahmand P., Popovic N., Claus P., LePrince P., Nicoletti A., Jalife J. (2015). Atrial fibrillation is associated with the fibrotic remodelling of adipose tissue in the subepicardium of human and sheep atria. Eur. Heart J..

[56] Mahajan R., Lau D.H., Brooks A.G., Shipp N.J., Manavis J., Wood J.P., Finnie J.W., Samuel C.S., Royce S.G., Twomey D.J. (2015). Electrophysiological, Electroanatomical, and Structural Remodeling of the Atria as Consequences of Sustained Obesity. J. Am. Coll. Cardiol..

[57] Golaszewska K., Harasim-Symbor E., Polak-Iwaniuk A., Chabowski A. (2019). Serum fatty acid binding proteins as a potential biomarker in atrial fibrillation. J. Physiol. Pharmacol..

[58] Iacobellis G., Bianco A. (2011). Epicardial adipose tissue: Emerging physiological, pathophysiological and clinical features. Trends Endocrinol. Metab..

[59] Venteclef N., Guglielmi V., Balse E., Gaborit B., Cotillard A., Atassi F., Amour J., Leprince P., Dutour A., Clément K. (2015). Human epicardial adipose tissue induces fibrosis of the atrial myocardium through the secretion of adipo-fibrokines. Eur. Heart J..

[60] Choromańska B., Myśliwiec P., Dadan J., Hady H., Chabowski A. (2011). The clinical significance of fatty acid binding proteins. Postep. Hig. Med. Dosw..

[61] Otaki Y., Watanabe T., Kubota I. (2017). Heart-type fatty acid-binding protein in cardiovascular disease: A systemic review. Clin. Chim. Acta.

[62] Shingu Y., Takada S., Yokota T., Shirakawa R., Yamada A., Ooka T., Katoh H., Kubota S., Matsui Y. (2020). Correlation between increased atrial expression of genes related to fatty acid metabolism and autophagy in patients with chronic atrial fibrillation. PLoS ONE.

[63] Vanlint S. (2013). Vitamin D and Obesity. Nutrients.

[64] Walsh J., Bowles S., Evans A.L. (2017). Vitamin D in obesity. Curr. Opin. Endocrinol. Diabetes Obes..

[65] Pereira-Santos M., Costa P.R.F., Assis A.M.O., Santos C.A.S.T., Santos D.B. (2015). Obesity and vitamin D deficiency: A systematic review and meta-analysis. Obes. Rev..

[66] Zhang X., Zhang S., Li Y., Detrano R.C., Chen K., Li X., Zhao L., Benjamin E.J., Wu Y. (2009). Association of obesity and atrial fibrillation among middle-aged and elderly Chinese. Int. J. Obes..

[67] Tedrow U.B., Conen D., Ridker P.M., Cook N.R., Koplan B.A., Manson J.E., Buring J.E., Albert C.M. (2010). The Long- and Short-Term Impact of Elevated Body Mass Index on the Risk of New Atrial Fibrillation: The WHS (Women’s Health Study). J. Am. Coll. Cardiol..

[68] Bellia A., Garcovich C., D’Adamo M., Lombardo M., Tesauro M., Donadel G., Gentileschi P., Lauro D., Federici M., Lauro R. (2011). Serum 25-hydroxyvitamin D levels are inversely associated with systemic inflammation in severe obese subjects. Intern. Emerg. Med..

[69] Gawałko M., Saljic A., Na Li N., Abu-Taha I., Jespersen T., Linz D., Nattel S., Heijman J., Fender A., Dobrev D. (2023). Adiposity-associated atrial fibrillation: Molecular determinants, mechanisms, and clinical significance. Cardiovasc. Res..

[70] Cabandugama P.K., Gardner M.J., Sowers J.R. (2016). The Renin Angiotensin Aldosterone System in Obesity and Hypertension: Roles in the Cardiorenal Metabolic Syndrome. Med. Clin. N. Am..

[71] Schütten M.T.J., Houben A.J.H.M., de Leeuw P.W., Stehouwer C.D.A. (2017). The Link Between Adipose Tissue Renin-Angiotensin-Aldosterone System Signaling and Obesity-Associated Hypertension. Physiology.

[72] (2022). Post-Operative Atrial Fibrillation: Current Treatments and Etiologies for a Persistent Surgical Complication. J. Surg. Res..

[73] Albini A., Malavasi V.L., Vitolo M., Imberti J.F., Marietta M., Lip G.Y., Boriani G. (2021). Long-term outcomes of postoperative atrial fibrillation following non cardiac surgery: A systematic review and metanalysis. Eur. J. Intern. Med..

[74] AbdelGawad A.M.E., Hussein M.A., Naeim H., Abuelatta R., Alghamdy S. (2019). A Comparative Study of TAVR versus SAVR in Moderate and High-Risk Surgical Patients: Hospital Outcome and Midterm Results. Heart Surg. Forum.

[75] Almassi G.H. (2015). Postoperative atrial fibrillation; the search goes on. J. Surg. Res..

[76] Da Silva R.G., De Lima G.G., Guerra N., Bigolin A.V., Petersen L.C. (2010). Risk Index Proposal to Predict Atrial Fibrillation after Cardiac Surgery. Rev. Bras. Cir. Cardiovasc..

[77] Dobrev D., Aguilar M., Heijman J., Guichard J.-B., Nattel S. (2019). Postoperative atrial fibrillation: Mechanisms, manifestations and management. Nat. Rev. Cardiol..

[78] Chen Y.-L., Zeng M., Liu Y., Xu Y., Bai Y., Cao L., Ling Z., Fan J., Yin Y. (2020). CHA2DS2-VASc Score for Identifying Patients at High Risk of Postoperative Atrial Fibrillation After Cardiac Surgery: A Meta-analysis. Ann. Thorac. Surg..

[79] Jacob K.A., Nathoe H.M., Dieleman J., van Osch D., Kluin J., Van Dijk D. (2014). Inflammation in new-onset atrial fibrillation after cardiac surgery: A systematic review. Eur. J. Clin. Investig..

[80] Barker T., May H.T., Doty J.R., Lappe D.L., Knowlton K.U., Carlquist J., Konery K., Inglet S., Chisum B., Galenko O. (2021). Vitamin D supplementation protects against reductions in plasma 25-hydroxyvitamin D induced by open-heart surgery: Assess-d trial. Physiol. Rep..

[81] McNally J.D., O’Hearn K., Lawson M.L., Maharajh G., Geier P., Weiler H., Redpath S., McIntyre L., Fergusson D., Menon K. (2015). Prevention of vitamin D deficiency in children following cardiac surgery: Study protocol for a randomized controlled trial. Trials.

[82] Gode S., Aksu T., Demirel A., Sunbul M., Gul M., Bakır I., Yeniterzi M. (2016). Effect of vitamin D deficiency on the development of postoperative atrial fibrillation in coronary artery bypass patients. J. Cardiovasc. Thorac. Res..

[83] Maesen B., Nijs J., Maessen J., Allessie M., Schotten U. (2011). Post-operative atrial fibrillation: A maze of mechanisms. Europace.

[84] Tønnesen R., Schwarz P., Hovind P., Jensen L.T. (2018). Modulation of the sympathetic nervous system in youngsters by vitamin-D supplementation. Physiol. Rep..

[85] Alexandre J., Saloux E., Chequel M., Allouche S., Ollitrault P., Plane A.-F., Legallois D., Fischer M.-O., Saplacan V., Buklas D. (2016). Preoperative plasma aldosterone and the risk of atrial fibrillation after coronary artery bypass surgery: A Prospective Cohort Study. J. Hypertens..

[86] Chequel M., Ollitrault P., Saloux E., Parienti J.-J., Fischer M.-O., Desgué J., Allouche S., Milliez P., Alexandre J. (2016). Preoperative Plasma Aldosterone Levels and Postoperative Atrial Fibrillation Occurrence Following Cardiac Surgery: A Review of Literature and Design of the ALDO-POAF Study (ALDOsterone for Prediction of Post-Operative Atrial Fibrillation). Curr. Clin. Pharmacol..

[87] Cerit L., Özcem B., Cerit Z., Duygu H. (2018). Preventive Effect of Preoperative Vitamin D Supplementation on Postoperative Atrial Fibrillation. Braz. J. Cardiovasc. Surg..

[88] Fan G., Liu J., Dong S., Chen Y. (2021). Postoperative Atrial Fibrillation after Minimally Invasive Direct Coronary Artery Bypass: A Single-Center, 5-Year Follow-Up Study. Heart Surg. Forum.

[89] Yaman B., Cerit L., Günsel H.K., Cerit Z., Usalp S., Yüksek Ü., Coşkun U., Duygu H., Akpınar O. (2020). Is There Any Link between Vitamin d and Recurrence of Atrial Fibrillation after Cardioversion?. Braz. J. Cardiovasc. Surg..

[90] Fakhry E.E., Ibrahim M.T. (2022). Relationship between vitamin D deficiency and success of cardioversion in patients with atrial fibrillation. Herzschrittmacherther. Elektrophysiol..

[91] Cerit L., Kemal H., Gulsen K., Ozcem B., Cerit Z., Duygu H. (2017). Relationship between Vitamin D and the development of atrial fibrillation after on-pump coronary artery bypass graft surgery. Cardiovasc. J. Afr..

[92] Emren S.V., Aldemir M., Ada F. (2016). Does Deficiency of Vitamin D Increase New Onset Atrial Fibrillation after Coronary Artery Bypass Grafting Surgery?. Heart Surg. Forum.

[93] Kara H., Yasim A. (2019). Effects of high-dose vitamin D supplementation on the occurrence of post-operative atrial fibrillation after coronary artery bypass grafting: Randomized controlled trial. Gen. Thorac. Cardiovasc. Surg..

[94] Ohlrogge A.H., Brederecke J., Ojeda F.M., Pecha S., Börschel C.S., Conradi L., Rimkus V., Blankenberg S., Zeller T., Schnabel R.B. (2022). The Relationship Between Vitamin D and Postoperative Atrial Fibrillation: A Prospective Cohort Study. Front. Nutr..

[95] Sahu M.K., Bipin C., Niraghatam H.V., Karanjkar A., Singh S.P., Rajashekar P., Ramakrishnan L., Devagourou V., Upadhyay A.D., Choudhary S.K. (2019). Vitamin D Deficiency and Its Response to Supplementation as “Stoss Therapy” in Children with Cyanotic Congenital Heart Disease Undergoing Open Heart Surgery. J. Card. Crit. Care TSS.

[96] Özsin K.K., Sanrı U.S., Toktaş F., Kahraman N., Yavuz Ş. (2018). Effect of Plasma Level of Vitamin D on Postoperative Atrial Fibrillation in Patients Undergoing Isolated Coronary Artery Bypass Grafting. Rev. Bras. Cir. Cardiovasc..

[97] Talasaz A.H., Salehiomran A., Heidary Z., Gholami K., Aryannejad H., Jalali A., Daei M. (2022). The effects of vitamin D supplementation on postoperative atrial fibrillation after coronary artery bypass grafting in patients with vitamin D deficiency. J. Card. Surg..

[98] Shadvar K., Ramezani F., Sanaie S., Maleki T.E., Arbat B.K., Nagipour B. (2016). Relationship between plasma level of vitamin D and post operative atrial fibrillation in patients undergoing CABG. Pak. J. Med. Sci..

[99] Daie M., Talasaz A.H., Karimi A., Gholami K., Salehiomran A., Ariannejad H., Jalali A. (2019). Relationship between Vitamin D Levels and the Incidence of Post Coronary Artery Bypass Graft Surgery Atrial Fibrillation. J. Tehran Univ. Heart Cent..

[100] Skuladottir G.V., Cohen A., Arnar D.O., Hougaard D.M., Torfason B., Palsson R., Indridason O.S. (2016). Plasma 25-hydroxyvitamin D_2_ and D_3_ levels and incidence of postoperative atrial fibrillation. J. Nutr. Sci..

[101] Ansari S.A., Dhaliwal J.S.S., Ansari Y., Ghosh S., Khan T.M.A. (2023). The Role of Vitamin D Supplementation Before Coronary Artery Bypass Grafting in Preventing Postoperative Atrial Fibrillation in Patients With Vitamin D Deficiency or Insufficiency: A Systematic Review and Meta-Analysis. Cureus.

[102] Das S., Bej P. (2022). Effect of Vitamin D Supplementation on Postoperative Outcomes in Cardiac Surgery Patients: A Systematic Review. J. Card. Crit. Care TSS.

[103] Liu X., Wang W., Tan Z., Zhu X., Liu M., Wan R., Hong K. (2019). The relationship between vitamin D and risk of atrial fibrillation: A dose-response analysis of observational studies. Nutr. J..

[104] Öztürk I. (2020). Atrial fibrillation after cardiac surgery and preoperative vitamin D levels: A systematic review and meta-analysis. Turk. J. Thorac. Cardiovasc. Surg..

[105] Rahimi M., Taban-Sadeghi M., Nikniaz L., Pashazadeh F. (2021). The relationship between preoperative serum vitamin D deficiency and postoperative atrial fibrillation: A systematic review and meta-analysis. J. Cardiovasc. Thorac. Res..

[106] Hameed I., Malik S., Nusrat K., Siddiqui O.M., Khan M.O., Mahmood S., Memon A., Usman M.S., Siddiqi T.J. (2023). Effect of vitamin D on postoperative atrial fibrillation in patients who underwent coronary artery bypass grafting: A systematic review and Meta-analysis. J. Cardiol..

[107] Acharya P., Safarova M.S., Dalia T., Bharati R., Ranka S., Vindhyal M., Jiwani S., Barua R.S. (2022). Effects of Vitamin D Supplementation and 25-Hydroxyvitamin D Levels on the Risk of Atrial Fibrillation. Am. J. Cardiol..

[108] Albert C.M., Cook N.R., Pester J., Moorthy M.V., Ridge C., Danik J.S., Gencer B., Siddiqi H.K., Ng C., Gibson H. (2021). Effect of Marine Omega-3 Fatty Acid and Vitamin D Supplementation on Incident Atrial Fibrillation: A Randomized Clinical Trial. JAMA.

[109] Qayyum F., Landex N.L., Agner B.R., Rasmussen M., Jøns C., Dixen U. (2012). Vitamin D deficiency is unrelated to type of atrial fibrillation and its complications. Dan. Med. J..

[110] Vitezova A., Cartolano N.S., Heeringa J., Zillikens M.C., Hofman A., Franco O.H., Jong J.C.K.-D. (2015). Vitamin D and the Risk of Atrial Fibrillation—The Rotterdam Study. PLoS ONE.

[111] Belen E., Aykan A., Kalay E., Sungur M., Sungur A., Çetin M. (2016). Low-Level Vitamin D Is Associated with Atrial Fibrillation in Patients with Chronic Heart Failure. Adv. Clin. Exp. Med..

[112] Boursiquot B.C., Larson J.C., Shalash O.A., Vitolins M.Z., Soliman E.Z., Perez M.V. (2019). Vitamin D with calcium supplementation and risk of atrial fibrillation in postmenopausal women. Am. Heart J..

[113] Smith M.B., May H.T., Blair T.L., Anderson J.L., Muhlestein J.B., Horne B.D., Lappe D.L., Day J.D., Crandall B.G., Weiss P. (2011). Abstract 14699: Vitamin D Excess Is Significantly Associated with Risk of Atrial Fibrillation. Circulation.

[114] Trevisan C., Piovesan F., Lucato P., Zanforlini B.M., De Rui M., Maggi S., Noale M., Corti M.C., Perissinotto E., Manzato E. (2019). Parathormone, vitamin D and the risk of atrial fibrillation in older adults: A prospective study. Nutr. Metab. Cardiovasc. Dis..

[115] Chen W.R., Liu Z.Y., Shi Y., Yin D.W., Wang H., Sha Y., Chen Y.D. (2013). Relation of Low Vitamin D to Nonvalvular Persistent Atrial Fibrillation in Chinese Patients. Ann. Noninvasive Electrocardiol..

[116] Rienstra M., Cheng S., Larson M.G., McCabe E.L., Booth S.L., Jacques P.F., Lubitz S.A., Yin X., Levy D., Magnani J.W. (2011). Vitamin D status is not related to development of atrial fibrillation in the community. Am. Heart J..

[117] Kavanagh M., Bradley E., Hoey L., Hughes C., McNulty H., Ward M., Strain J., Tracey F., Molloy A., Laird E. (2022). 51 vitamin D deficiency is associated with increased risk of atrial fibrillation: A cross-sectional analysis. Age Ageing.

[118] Thompson J., Nitiahpapand R., Bhatti P., Kourliouros A. (2015). Vitamin D deficiency and atrial fibrillation. Int. J. Cardiol..

[119] Zhang Z., Yang Y., Ng C.Y., Wang D., Wang J., Li G., Liu T. (2016). Meta-analysis of Vitamin D Deficiency and Risk of Atrial Fibrillation. Clin. Cardiol..

[120] Bie L. (2019). The Status and Research Progress on Vitamin D Deficiency and Atrial Fibrillation. Braz. J. Cardiovasc. Surg..

[121] Cosentino N., Campodonico J., Milazzo V., De Metrio M., Brambilla M., Camera M., Marenzi G. (2021). Vitamin D and Cardiovascular Disease: Current Evidence and Future Perspectives. Nutrients.

[122] Morillo C.A., Banerjee A., Perel P., Wood D., Jouven X. (2017). Atrial Fibrillation: The Current Epidemic. J. Geriatr. Cardiol. JGC.

[123] Bouillon R., Carmeliet G., Verlinden L., van Etten E., Verstuyf A., Luderer H.F., Lieben L., Mathieu C., Demay M. (2008). Vitamin D and Human Health: Lessons from Vitamin D Receptor Null Mice. Endocr. Rev..

[124] Lips P. (2006). Vitamin D Physiology. Prog. Biophys. Mol. Biol..

[125] Norman A.W. (2006). Vitamin D Receptor: New Assignments for an Already Busy Receptor. Endocrinology.

[126] Wang Y., Zhu J., DeLuca H.F. (2012). Where Is the Vitamin D Receptor?. Arch. Biochem. Biophys..

[127] Bhattarai H.K., Shrestha S., Rokka K., Shakya R. (2020). Vitamin D, Calcium, Parathyroid Hormone, and Sex Steroids in Bone Health and Effects of Aging. J. Osteoporos..

[128] Khundmiri S.J., Murray R.D., Lederer E. (2011). PTH and Vitamin D. Comprehensive Physiology.

[129] Saraiva G.L., Cendoroglo M.S., Ramos L.R., Araújo L.M.Q., Vieira J.G.H., Kunii I., Hayashi L.F., Corrêa M.d.P., Lazaretti-Castro M. (2005). Influence of ultraviolet radiation on the production of 25 hydroxyvitamin D in the elderly population in the city of São Paulo (23 o 34’S), Brazil. Osteoporos. Int..

[130] Lugg S.T., Howells P.A., Thickett D.R. (2015). Optimal Vitamin D Supplementation Levels for Cardiovascular Disease Protection. Dis. Markers.

[131] Acharya P., Dalia T., Ranka S., Sethi P., Oni O.A., Safarova M.S., Parashara D., Gupta K., Barua R.S. (2021). The Effects of Vitamin D Supplementation and 25-hydroxyvitamin D Levels on The Risk of MI and Mortality. J. Endocr. Soc..

[132] Maurya V.K., Aggarwal M. (2017). Factors influencing the absorption of vitamin D in GIT: An overview. J. Food Sci. Technol..

[133] Tripkovic L., Lambert H., Hart K., Smith C.P., Bucca G., Penson S., Chope G., Hyppönen E., Berry J., Vieth R. (2012). Comparison of vitamin D2 and vitamin D3 supplementation in raising serum 25-hydroxyvitamin D status: A systematic review and meta-analysis. Am. J. Clin. Nutr..

[134] Hollander D., Muralidhara K.S., Zimmerman A. (1978). Vitamin D-3 intestinal absorption in vivo: Influence of fatty acids, bile salts, and perfusate pH on absorption. Gut.

[135] Compston J.E., Merrett A.L., Hammett F.G., Magill P. (1981). Comparison of the Appearance of Radiolabelled Vitamin D3 and 25-Hydroxy-Vitamin D3 in the Chylomicron Fraction of Plasma after Oral Administration in Man. Clin. Sci..

[136] Bennour I., Haroun N., Sicard F., Mounien L., Landrier J.-F. (2022). Vitamin D and Obesity/Adiposity—A Brief Overview of Recent Studies. Nutrients.

[137] Li Y., Chen C., Liu H.L., Qian G. (2015). Vitamin D, Parathyroid Hormone, and Heart Failure in A Chinese Elderly Population. Endocr. Pract..

[138] Ding F., Nie X., Li X., He Y., Li G. (2021). Data mining: Biological and temporal factors associated with blood parathyroid hormone, vitamin D, and calcium concentrations in the Southwestern Chinese population. Clin. Biochem..

[139] Taheri M., Tavasoli S., Shokrzadeh F., Amiri F.B., Basiri A. (2019). Effect of vitamin D supplementation on 24-hour urine calcium in patients with calcium Urolithiasis and vitamin D deficiency. Int. Braz. J. Urol..

[140] Fisher A., Srikusalanukul W., Fisher L., Smith P.N. (2022). Comparison of Prognostic Value of 10 Biochemical Indices at Admission for Prediction Postoperative Myocardial Injury and Hospital Mortality in Patients with Osteoporotic Hip Fracture. J. Clin. Med..

[141] Hanel A., Carlberg C. (2020). Skin colour and vitamin D: An update. Exp. Dermatol..

[142] Allan V., Honarbakhsh S., Casas J.-P., Wallace J., Hunter R., Schilling R., Perel P., Morley K., Banerjee A., Hemingway H. (2017). Are cardiovascular risk factors also associated with the incidence of atrial fibrillation?. Thromb. Haemost..

[143] Gutierrez C., Blanchard D.G. (2011). Atrial fibrillation: Diagnosis and treatment. Am. Fam. Physician.

[144] Benedetto U., Gaudino M.F., Dimagli A., Gerry S., Gray A., Lees B., Flather M., Taggart D.P., ART Investigators (2020). Postoperative Atrial Fibrillation and Long-Term Risk of Stroke After Isolated Coronary Artery Bypass Graft Surgery. Circulation.

